# Beyond GWAS: Investigating Structural Variants and Their Segregation in Familial Alzheimer’s Disease

**DOI:** 10.1002/alz.092677

**Published:** 2025-01-09

**Authors:** Tamil Iniyan Gunasekaran, Dolly Reyes‐Dumeyer, André Corvelo, Wayne E. Clarke, Uday S. Evani, Marta S. Byrska‐Bishop, Anna O. Basile, Alexi Runnels, Rajeeva O. Musunuri, Giuseppe Narzisi, Kelley M. Faber, Alison M. Goate, Brad F. Boeve, Carlos Cruchaga, Margaret A. Pericak‐Vance, Jonathan L. Haines, Roger N Rosenberg, Debby W Tsuang, Diones Rivera Mejia, Martin Medrano, Rafael A. Lantigua, Robert Sweet, David A. Bennett, Robert S. Wilson, Tatiana M. Foroud, Clifton L. Dalgard, Richard Mayeux, Michael Zody, Badri N Vardarajan

**Affiliations:** ^1^ The Gertrude H. Sergievsky Center, College of Physicians and Surgeons, Columbia University, New York, NY USA; ^2^ The Taub Institute for Research on Alzheimer’s Disease and the Aging Brain, Vagelos College of Physicians & Surgeons, Columbia University, New York, NY USA; ^3^ Department of Neurology, Vagelos College of Physicians and Surgeons, Columbia University, and the New York Presbyterian Hospital, New York, NY USA; ^4^ New York Genome Center, New York, NY USA; ^5^ National Centralized Repository for Alzheimer's Disease and Related Dementias (NCRAD), Indianapolis, IN USA; ^6^ Indiana University School of Medicine, Indianapolis, IN USA; ^7^ Department of Medical and Molecular Genetics, Indiana University School of Medicine, Indianapolis, IN USA; ^8^ Ronald M. Loeb Center for Alzheimer's Disease, Friedman Brain Institute, Icahn School of Medicine at Mount Sinai, New York, NY USA; ^9^ Department of Genetics and Genomic Sciences, Icahn School of Medicine at Mount Sinai, New York, NY USA; ^10^ Department of Neurology, Mayo Clinic, Rochester, MN USA; ^11^ Department of Psychiatry, Washington University School of Medicine, St. Louis, MO USA; ^12^ John P. Hussman Institute for Human Genomics, Miller School of Medicine, Miami, FL USA; ^13^ Case Western Reserve University School of Medicine, Department of Population & Quantitative Health Sciences, Cleveland Institute for Computational Biology, Cleveland, OH USA; ^14^ Department of Neurology, UTSouthwestern Medical Center Dallas, Dallas, TX USA; ^15^ Geriatric Research, Education, and Clinical Center, VA Puget Sound Health Care System, Seattle, WA USA; ^16^ University of Washington, Seattle, WA USA; ^17^ CEDIMAT, Santo Domingo, Santo Domingo Dominican Republic; ^18^ Universidad Pedro Henríquez Urena, Santo Domingo Dominican Republic; ^19^ Pontificia Universidad Catolica Madre y Maestra, Santiago Dominican Republic; ^20^ Department of Medicine, Vagelos College of Physicians & Surgeons, Columbia University, New York, NY USA; ^21^ Department of Psychiatry and Neurology, University of Pittsburgh, Pittsburgh, PA USA; ^22^ Rush Alzheimer's Disease Center, Rush University Medical Center, Chicago, IL USA; ^23^ Indiana University School of Medicine/NCRAD, Indianapolis, IN USA; ^24^ Department of Anatomy, Physiology, and Genetics, Uniformed Services University of the Health Sciences, Bethesda, MD USA; ^25^ Taub Institute for Research on Alzheimer’s Disease and the Aging Brain, Columbia University Medical Center, New York, NY USA; ^26^ Columbia University Medical Center, New York, NY USA; ^27^ Taub Institute for Research on Alzheimer’s Disease and the Aging Brain, Columbia University, New York, NY USA; ^28^ Department of Neurology, Columbia University, New York, NY USA

## Abstract

**Background:**

Late‐Onset Alzheimer’s Disease (LOAD) is characterized by genetic heterogeneity and there is no single model explaining the genetic mode of inheritance. To date, more than 70 genetic loci associated with AD have been identified but they explain only a small proportion of AD heritability. Structural variants (SVs) may explain some of the missing AD heritability, and specifically, their segregation in AD families has yet to be investigated.

**Method:**

We analyzed WGS data from 197 NHW families (926 subjects, 58.5% affected) and 214 CH families (1,340 subjects, 59.17% affected). Manta, Absinthe, and MELT were used for large insertions/deletions calling from short‐read WGS, combined with Sniffles2 calls from 4 ONT‐sequenced genomes and an external SV call set from HGSVC on 32 PacBio‐sequenced genomes from the 1000 Genomes Project. Genotyping produced a unified project‐level VCF. We identified 45,251 insertions and 76,566 deletions genome‐wide. Variants were tested for segregation and pathogenicity using Annot‐SV, cadd‐SV, and Variant Effect Predictor. Segregation required SV presence in all affected family members and only in unaffected members five years younger than average disease onset.

**Result:**

We identified 453 insertions and 598 deletions segregating in 78.68% and 87.31% of NHW families, respectively. In CH families, 432 insertions and 460 deletions were segregating in 75.23% and 72.90% of the families, respectively. Genes overlapping with the SVs exhibited high expression levels in brain tissues. Notably, around 93% of insertions and 76% of deletions segregating in NHW and CH families were less than 1 kilobase pair (1kbp) in length. A total of 79 insertions and 96 deletions were found to be segregating in both NHW and CH families. Interestingly, a segregating insertion was observed in CH families overlapping within the CACNA2D3 gene, which was previously reported in a CH GWAS for clinical AD. A deletion segregating in NHW overlapped with the PSEN1, and another in a CH family overlapped with the PTK2B gene.

**Conclusion:**

Our findings suggested that there are several SVs associated with familial AD across CH and NHW families. Prioritizing the SVs based on their effects on gene function and expression will be helpful in understanding their contributions in AD.

